# Maternal exercise upregulates mitochondrial gene expression and increases enzyme activity of fetal mouse hearts

**DOI:** 10.14814/phy2.13184

**Published:** 2017-03-14

**Authors:** Eunhee Chung, Hayli E. Joiner, Tracer Skelton, Kalli D. Looten, Maria Manczak, P. Hemachandra Reddy

**Affiliations:** ^1^ Department of KinesiologyHealth, and NutritionUniversity of Texas at San AntonioSan AntonioTexas; ^2^ Department of Kinesiology and Sport ManagementTexas Tech UniversityLubbockTexas; ^3^ Cell Biology and Biochemistry and Garrison Institute on AgingTexas Tech University Health Sciences CenterLubbockTexas

**Keywords:** Cytochrome c oxidase, electron transport chain, fetal hearts, hydrogen peroxide, mitochondria

## Abstract

Maternal exercise during pregnancy has been shown to improve the long‐term health of offspring in later life. Mitochondria are important organelles for maintaining adequate heart function, and mitochondrial dysfunction is linked to cardiovascular disease. However, the effects of maternal exercise during pregnancy on mitochondrial biogenesis in hearts are not well understood. Thus, the purpose of this study was to test the hypothesis that mitochondrial gene expression in fetal myocardium would be upregulated by maternal exercise. Twelve‐week‐old female C57BL/6 mice were divided into sedentary and exercise groups. Mice in the exercise group were exposed to a voluntary cage‐wheel from gestational day 1 through 17. Litter size and individual fetal weights were taken when pregnant dams were sacrificed at 17 days of gestation. Three to four hearts from the same group were pooled to study gene expression, protein expression, and enzyme activity. There were no significant differences in litter size, sex distribution, and average fetal body weight per litter between sedentary and exercised dams. Genes encoding mitochondrial biogenesis and dynamics, including nuclear respiratory factor‐1 (*Nrf1*), *Nrf2*, and dynamin‐related GTPase termed mitofusin‐2 (*Mfn2*) were significantly upregulated in the fetal hearts from exercised dams. Cytochrome c oxidase activity and ATP production were significantly increased, while the hydrogen peroxide level was significantly decreased in the fetal hearts by maternal exercise. Our results demonstrate that maternal exercise initiated at day 1 of gestation could transfer the positive mitochondrial phenotype to fetal hearts.

## Introduction

The Developmental Origins of Health and Disease (DOHD) paradigm suggests that the health of offspring is determined as early as fetal status, and the intrauterine environment is an important indicator of the health of offspring in later life (Schulz 2010). Ample studies have demonstrated that an adverse intrauterine environment or maternal behavior, including placental restriction, maternal obesity, and smoking, not only negatively influence mothers' health but also produce a negative environment for fetal development and predispose offspring to disease later in life (Hanson and Gluckman 2014; Segovia et al. 2014). On the other hand, positive maternal behavioral modification through maternal exercise before and during pregnancy leads to improved fetal heart function, including lower fetal heart rate, increased heart rate variability (May et al. 2010), and reduced risk of congenital heart disease in offspring (Schulkey et al. 2015). These studies suggest that the beneficial effects of exercise on the heart can be seen as early as the gestational developmental period.

Mitochondria are the critical sites of energy sensing and adenosine triphosphate (ATP) production via oxidative phosphorylation (OXPHOS) in concurrence with Krebs' cycle and *β*‐oxidation of fatty acids (Bhatti et al. 2016). Consequently, mitochondria are important organelles for maintaining adequate heart function since cardiac muscle requires a constant energy supply (Rimbaud et al. 2009). It is becoming apparent that alterations in mitochondrial number and morphology in the heart play important roles in determining cardiac function (Rimbaud et al. 2009; Dorn et al. 2015; Verma et al. 2016). Remarkably, mitochondria undergo structural and functional changes during early‐ to mid‐gestation when oxygen becomes more available to the fetus (Alcolea et al. 2006; Minai et al. 2008; Pejznochova et al. 2010).

The term mitochondrial biogenesis refers to the process of mitochondrial proliferation including growth and division of preexisting mitochondrion (Ventura‐Clapier et al. 2008; Rimbaud et al. 2009). Mitochondria also undergo structural and morphological changes through the process of fusion and fission, called mitochondrial dynamics (Rimbaud et al. 2009). The transcriptional coactivator peroxisome proliferator‐activated receptor‐*γ* coactivator‐1*α* (PGC‐1*α*), known as a master regulator of mitochondrial biogenesis, plays a role in controlling the rate of mitochondrial biogenesis by activating different transcription factors including nuclear respiratory factor (NRF1) and NRF2. NRF1 and NRF2 further activate mitochondrial transcription factor A (TFAM), which promotes mitochondrial‐encoded gene transcription. Mitochondrial fusion is regulated by mitofusin 1 (MFN1), MFN2, and optic atrophy 1 (OPA1), while fission is regulated by fission 1(FIS1) and dynamic‐related protein (Drp1). Disturbed mitochondrial dynamics and altered mitochondrial biogenesis directly impact mitochondrial function, resulting in an excessive generation of reactive oxygen species, altered mitochondrial enzymatic activities, diminished ATP production, and impaired energy metabolism (Bhatti et al. 2016); thus could ultimately lead to cardiac dysfunction (Suematsu et al. 2003).

Regular exercise has been shown to trigger many pathways involving mitochondrial biogenesis and dynamics in the heart (Jiang et al. 2014; Vettor et al. 2014). However, little is known about how maternal exercise influences fetal mitochondrial biogenesis and dynamics on developing fetal hearts. Thus, the aim of the current study was to test the hypothesis that mitochondrial gene expression in fetal myocardium would be upregulated by maternal exercise. Herein, we showed that mitochondrial biogenesis in fetal myocardium is mainly increased at transcriptional levels, with increased cytochrome c oxidase activity, the rate‐limiting enzyme of electron transport chain, and ATP production in response to maternal exercise.

## Materials and Methods

### Animals

Virgin, female C57BL/6 mice were obtained from Charles River Laboratories (Wilmington, MA) at 10 weeks of age. Animals were housed in a temperature‐ and light‐controlled room and fed a standard laboratory chow diet (2020X Teklad Global Soy Protein‐Free Extruded Rodent Diet, Harlan). Food and water were available *ad libitum* throughout the study. All of the animals were handled and euthanized under the approved protocol by the University Animal Care and Use Committee.

Five mice were housed per cage for a minimum of 1‐week acclimation. After the acclimation period, mice were randomly divided into two groups, sedentary and exercise during pregnancy. The stage of the estrus cycle was evaluated by the appearance of the vagina (Champlin et al. 1973) as used in previous studies (Chung et al. 2012, 2013) for 1 week prior to initiating mating. At 12 weeks of age, when female mice were in the proestrus phase of their cycle, a 10‐week‐old male C57BL/6 proven breeder mouse was added to the female cage; breeding was performed in harems: four male mice were rotationally used to avoid potential sire effect since male mice can be reused. The male mice were housed individually to avoid territory fighting and fed the same diet as female mice. The male mouse was removed once a copulatory plug was detected, and this day was considered as day one of pregnancy; birth most often occurs at day 20 (Chung et al. 2012). Since the paternal programming effects on fetal development have been documented (Ng et al. 2010; Mcpherson et al. 2015), the male mouse was removed right after confirming pregnancy. Mice in the sedentary group who became pregnant on the same day were housed together in a cage (typically 3 per cage). Pregnant dams in the exercise group were housed individually in order to monitor exercise volume, and they exercised voluntarily from gestational day 1 to the morning of gestational day 17. The detailed information regarding voluntary wheels and cages used for this study were described previously (Allen et al. 2001). The running distance and duration were recorded daily with a bicycle odometer (model BC 600, Sigma Sport, Olney, IL). All pregnant dams were sacrificed at gestational day 17 (3 days before parturition). To determine the role of exercise during pregnancy on fetal outcomes, fetal body weight (FW) and the number of fetuses per dam were recorded on the day of tissue collection. Average FW was obtained by averaging fetal body weight per litter and then taking each litter mean to get the mean and standard error of the mean (SEM) of each group as described previously (Gilbert et al. 2012; Carter et al. 2013).

To avoid the effects of acute exercise and feeding, all pregnant dams were fasted for 4 h in a sedentary cage before being euthanized. Pregnant dams were euthanized by placing the mouse in an isoflurane‐induction machine followed by cervical dislocation. Immediately, fetal heart tissues were collected and stored at −80°C for further analyses.

### mRNA analysis

Three to four fetal hearts within each group were pooled to study gene expression. Total RNA was isolated using a Tri Reagent kit according to the manufacturer's instructions (Molecular Research Center, Inc., Cincinnati, OH). One microgram of total RNA was used to synthesize cDNA with the iScript Reverse Transcription Supermix for a quantitative reverse transcription PCR (RT‐qPCR) kit (BioRad) with random primers according to the manufacturer's instructions. Gene expression was determined by RT‐qPCR using SYBR Green dye with gene specific primer sets and a BioRad CFX real‐time system. The cycle threshold (CT) values of all candidate genes were normalized to *β*‐actin, a reference gene, and the ∆CT values were calculated. The results were plotted as fold changes relative to the sedentary group. Primers used for this study are listed in Table 1. mRNAs were written according to the guidelines for formatting mouse gene name (http://www.biosciencewriters.com/Guidelines-for-Formatting-Gene-and-Protein-Names.aspx): italicized with only the first letter in upper‐case (i.e., *Nrf1*). Protein symbols of mice are not italicized and all letters are in upper case (i.e., NRF1).

**Table 1:** Summary of qRT‐PCR oligonucleotide primers used in measuring mRNA expression of mitochondrial biogenesis, dynamics, and electron transport chains

Gene	DNA sequence (5′‐3′)	PCR product size
Mitochondrial biogenesis		
*Ppargc1a*	Forward Primer GCAGTCGCAACATGCTCAAG	83
	Reverse Primer GGGAACCCTTGGGGTCATTT	
*Tfam*	Forward Primer TCCACAGAACAGCTACCCAA	84
	Reverse Primer CCACAGGGCTGCAATTTTCC	
*Nrf1*	Forward Primer AGAAACGGAAACGGCCTCAT	96
	Reverse Primer CATCCAACGTGGCTCTGAGT	
*Nrf2*	Forward Primer ATGGAGCAAGTTTGGCAGGA	96
	Reverse Primer GCTGGGAACAGCGGTAGTAT	
Mitochondrial dynamics		
*Drp1*	Forward Primer ATGCCAGCAAGTCCACAGAA	86
	Reverse Primer TGTTCTCGGGCAGACAGTTT	
*Fis1*	Forward Primer CAAAGAGGAACAGCGGGACT	95
	Reverse Primer ACAGCCCTCGCACATACTTT	
*Mfn1*	Forward Primer GCAGACAGCACATGGAGAGA	83
	Reverse Primer GATCCGATTCCGAGCTTCCG	
*Mfn2*	Forward Primer TGCACCGCCATATAGAGGAAG	78
	Reverse Primer TCTGCAGTGAACTGGCAATG	
*Opa1*	Forward Primer ACCTTGCCAGTTTAGCTCCC	82
	Reverse Primer TTGGGACCTGCAGTGAAGAA	
*CypD*	Forward Primer AGATGTCAAATTGGCAGGGGG	91
	Reverse Primer TGCGCTTTTCGGTATAGTGCT	
Mitochondrial‐encoded Electron Transport Chain Genes		
*ND1* – CI	Forward Primer ATTACTTCTGCCAGCCTGACC	70
	Reverse Primer GGCCCGGTTTGTTTCTGCTA	
*ND5* – CI	Forward Primer CGATGTCTCCGATGCGGTTA	71
	Reverse Primer GAAGGAGGGATTGGGGTAGC	
*Cytb –* CIII	Forward Primer GGCTACGTCCTTCCATGAGG	75
	Reverse Primer TGGGATGGCTGATAGGAGGT	
*COX1 –* CIV	Forward Primer ATCACTACCAGTGCTAGCCG	84
	Reverse Primer CCTCCAGCGGGATCAAAGAA	
*ATP6 –* CV	Forward Primer TCCCAATCGTTGTAGCCATCA	76
	Reverse Primer AGACGGTTGTTGATTAGGCGT	
Housekeeping genes		
*Actb*	Forward Primer AGAAGCTGTGCTATGTTGCTCTA	91
	Reverse Primer TCAGGCAGCTCATAGCTCTTC	

*Actb*, beta actin; *Drp1*, dynamic‐related protein 1; *Fis1*, fission 1; *Mfn*, dynamin‐related GTPase termed mitofusin; *Atp6,* ATP synthase 6; *Co 1*, mitochondria‐encoded cytochrome c oxidase, COX1; *Cytb,* mitochondria‐encoded cytochrome B; *CypD*, peptidylprolyl isomerase D; *Nd1,* mitochondria‐encoded NADH dehydrogenase 1; *Nd5,* mitochondrial NADH dehydrogenase 5; *Nrf*, nuclear respiratory factor; *Opa1*, Optic atrophy protein 1; *Ppargc1a*, peroxisome proliferator‐activated receptor gamma coactivator 1‐ alpha; *Tfam*, mitochondrial transcription factor A.

### Protein analyses

Three to four fetal hearts within each group were pooled to measure protein expression levels and enzyme activity assays. Fetal hearts were homogenized in an ice‐cold lysis buffer (Pierce IP lysis buffer: 25 mmol/L Tris∙HCl pH 9.4, 150 mmol/L NaCl, 1% NP‐40, 1 mmol/L EDTA, and 5% glycerol) containing protease and phosphatase inhibitor single‐use cocktail (Thermo Scientific), centrifuged at 10,000 g for 10 min at 4°C, and the supernatant was transferred to a tube, and stored at −80°C for further analyses. Total protein concentration was determined using a BCA protein assay kit according to the manufacturer's instructions (Thermo Scientific). Detailed information about the western blot method was described previously (Chung et al. 2012) with modifications regarding imaging and data analyses. Briefly, ponceau staining was used to assure an equal amount of protein loading and transfer efficiency. All images were taken using the ChemiDoc MP system (BioRad) and the intensity of bands was quantified using Image Lab software (BioRad). The following antibodies were used in the present study: anti‐NRF1 (ab86516; Abcam), anti‐NRF2 (NBP1‐32822; Novus Biologicals), anti‐PGC‐1*α* (NBPI‐04676; Novus Biologicals), anti‐MFN1 (NBP1‐51841; Novus biological), anti‐MFN2 (12186‐1‐AB; Proteintech), total OXPHOS rodent WB antibody cocktail (ab110413; Abcam), and anti‐GAPDH (2118; cell signaling). For western blot of total OXPHOS, rat liver mitochondria provided by Abcam (ab110413) was loaded together with samples as a positive control since the cocktail contained five different OXPHOS proteins. Western blot analyses were not performed for all mitochondrial proteins because of lack of sufficient tissues.

### Assays

The production of hydrogen peroxide (H_2_O_2_) and ATP in the fetal hearts were measured using an Amplex Red H_2_O_2_ assay kit (A22188; Invitrogen) and the ATP determination kit (A22066; Invitrogen) as previously described (Manczak et al. 2006). Cytochrome c oxidase activity was determined by a colorimetric assay kit (CYTOCOX1; Sigma‐Aldrich). All assays were done according to the manufacturer's instruction. Briefly, 10 *μ*g of protein from homogenized samples was used to determine H_2_O_2_ level, ATP production, and cytochrome c oxidase activity. The values were reported after subtracting the background, determined using a reaction without tissue.

### Statistical analysis

All results are expressed as mean ± SEM. Statistical significance was tested by Student's t‐test to compare group differences. A value of *P* ≤ 0.05 was regarded as significant between groups. Statistical analyses were performed using GraphPad Prism version 6 software.

## Results

### Phenotypes of pregnant dams

**Figure 1:**
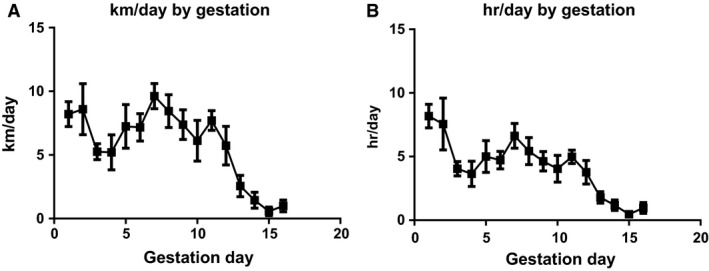
Maternal exercise during pregnancy. **(**A) Total running distance per day (km/day) and (B) Total duration per day (hr/day) gradually decreased as pregnancy progressed. Values are expressed as mean ± SEM. *n* = 5 for sedentary pregnant dams and *n* = 8 for exercised pregnant dams.

The pattern of running activity was high in early pregnancy and gradually decreased as pregnancy progressed (Fig. 1A and B). We recorded body weight before initiation of pregnancy and right before tissue collection to determine the influence of exercise on gestational body weight gain (Table 2). As shown in Table 2, there was no significant difference in gestational weight gain between groups (*P* = 0.24). Maternal exercise did not affect fetal growth, as there was no significant difference seen in the number of fetuses and average fetal weight per dams (*P* = 0.26) between groups. Further, sex distribution was not different (*P* = 0.44) between sedentary and exercised groups (Table 2).

**Table 2:** Characteristics of pregnant dams and fetuses

	Sedentary (*n* = 5)	Exercised (*n* = 8)
BW‐before pregnancy (g)	22.9 ± 0.5	23.0 ± 0.3
BW‐final (g)	34.3 ± 1.3	33.7 ± 0.6
GWG	16.0 ± 1.1	14.5 ± 0.6
Average FBW (mg)/dams	558.1 ± 11.9	608.5 ± 28.6
# pups/dams	8.2 ± 1.3	8.0 ± 0.5
% male/dams	54.4 ± 12.2	41.6 ± 10.3

Values are expressed as mean ± standard error of mean (SEM). n, number of mice per group. BW, body weight; FBW, fetal body weight; GWG, gestational weight gain.

### Mitochondrial biogenesis in fetal myocardium

**Figure 2:**
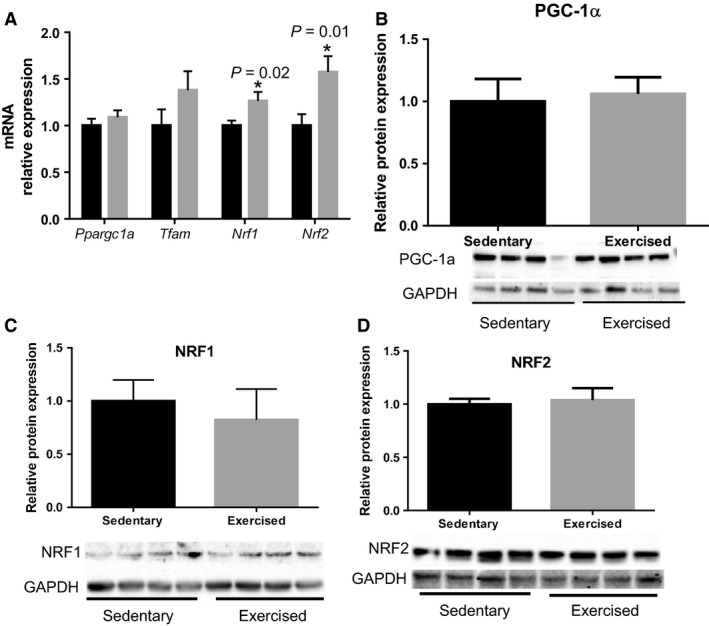
Maternal exercise during pregnancy on mitochondrial biogenesis in the fetal hearts. (A) Levels of relative mRNA expression measured by qRT‐PCR. *n* = 9–12/group. Maternal exercise during pregnancy did not alter levels of mRNA in *Ppargc1a* and *Tfam*, while it significantly upregulated the levels of mRNA in *Nrf1* and *Nrf2*. (B–D) Densitometric analyses of protein expression levels relative to the sedentary group with representative images of western blots were shown. No significant differences in PGC‐1*α*, NRF1, and NRF2 (*P* > 0.05). *n* = 5–6/group. * *P* < 0.05, significantly different from the sedentary group. Black bar: fetal hearts from sedentary dams; gray bar: fetal hearts from exercised dams.

To investigate the role of maternal exercise during pregnancy on mitochondrial biogenesis in the fetal heart, we determined mRNA levels of genes regulating mitochondrial biogenesis: *Ppargc1a*,* Tfam*,* Nrf1*, and *Nrf2*. *Ppargc1a* (*P* = 0.42) and *Tfam* (*P* = 0.17) were not different, while *Nrf1* (*P* = 0.02) and *Nrf2* (*P* = 0.01) were significantly increased in the fetal hearts from exercised dams compared to sedentary dams (Fig. 2A). However, we did not find changes in PGC‐1*α*, NRF1, and NRF2 protein expression between groups (Fig. 2B–D).

### Mitochondrial dynamics genes in fetal myocardium

**Figure 3:**
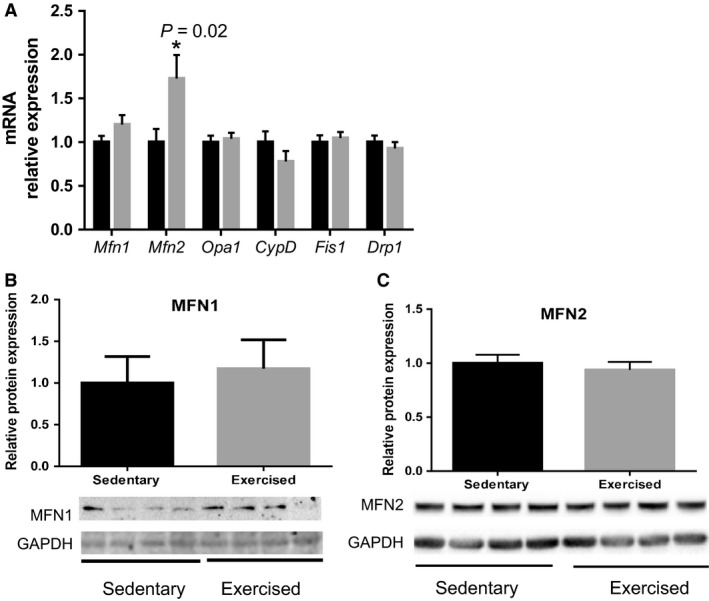
Maternal exercise during pregnancy on mitochondrial dynamics in the fetal hearts. (A**)** Relative mRNA expression measured by qRT‐PCR. *n* = 9–12/group. Maternal exercise significantly increased *Mfn2*, but other dynamic genes were not changed. (B–C) Densitometric analyses of protein expression levels relative to the sedentary group with representative images of western blot were shown. *n* = 5–6/group. Values are expressed by mean ± SEM expressed as fold change relative to the sedentary group. * *P* < 0.05, significantly different from the sedentary group. Black bar: fetal hearts from sedentary dams; gray bar: fetal hearts from exercised dams.

Mitochondria are dynamic organelles which are capable of changing their morphology by fusion and fission (Fig. 3). We measured mRNA levels of genes (Fig. 3A) regulating mitochondrial dynamics: *Mfn1*,* Mfn2*,* Opa1*,* Drp1*,* Fis1*, and peptidylprolyl isomerase D (known as cyclophilin D (*CypD*)). Only *Mfn2* was significantly increased in the fetal hearts from exercised pregnant dams compared to sedentary pregnant dams (*P* = 0.02). Similar to genes regulating mitochondrial biogenesis, increased mRNA level of *Mfn2* did not induce protein levels (Fig. 3C).

### Mitochondrial respiratory chain complex genes and proteins in fetal myocardium

**Figure 4:**
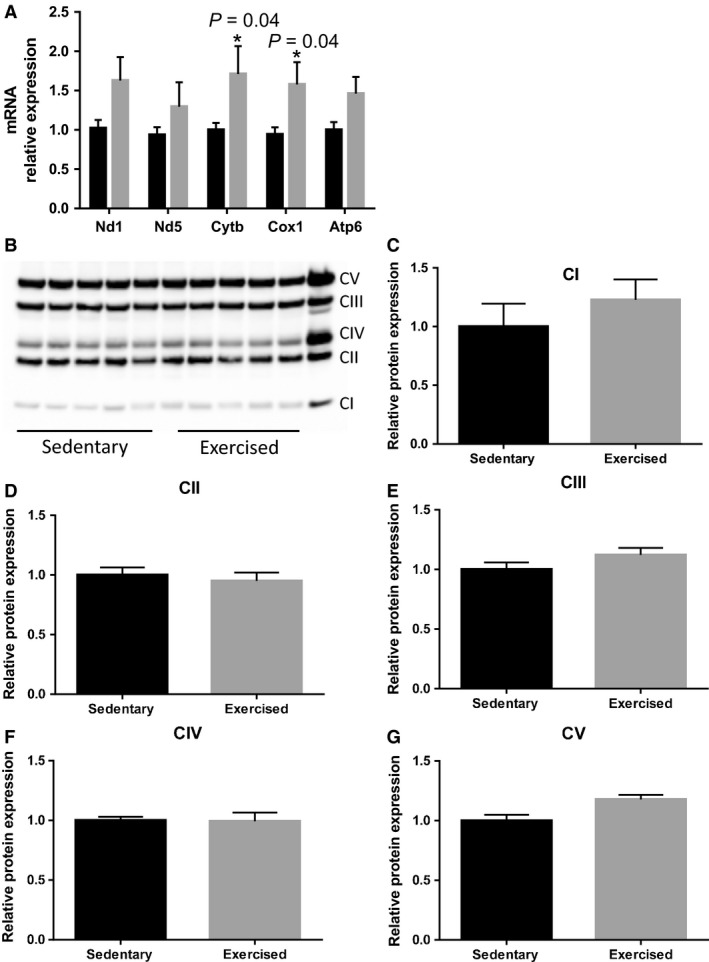
Maternal exercise during pregnancy on mitochondrial respiratory chain gene and oxidative phosphorylation complexes in the fetal hearts. (A) Relative expression of genes encoding respiratory chain complexes (I, III, IV, and V) measured by qRT‐PCR. *n* = 9–12/group. Complex I, ND subunit (*Nd1*) did not reach the level of statistical significance (*P* = 0.06) and *Nd5* was not significantly increased (*P* = 0.17). Maternal exercise significantly increased complex III, mitochondria‐encoded NADH dehydrogenase I (*Cytb*) and complex IV, mitochondria‐encoded cytochrome c oxidase I (*Cox1*) in the fetal hearts. Complex V, mitochondria‐encoded ATP synthase 6 (*Atp6*) did not reach the level of statistical significances (*P* = 0.06). B) Representative western blot images of complex I (CI) to CV. Rat heart mitochondrial western blot control (provided by Abcam) was loaded (right end) with samples as a positive control. (C–G) Densitometric analyses showed no significant effect of maternal exercise on oxidative phosphorylation complex proteins. *n* = 5 per group. Values are expressed as mean ± SEM expressed as fold change relative to the sedentary group. * *P* < 0.05, significantly different from the sedentary group. Black bar: fetal hearts from sedentary dams; gray bar: fetal hearts from exercised dams.

We measured key genes encoding subunits of the electron transport chain, where the major energy generation occurs through oxidative phosphorylation. As shown in Figure 4A, mitochondrial NADH dehydrogenase 1, complex I (*Nd1*), mitochondrial NADH dehydrogenase 5, complex 1 (*Nd5*), cytochrome b, complex III (*Cytb*), mitochondrial cytochrome c oxidase I, complex IV (*Cox1*), and mitochondrial ATP synthase 6, complex V (*Atp6*) all showed an increasing trend (i.e., *Nd1* and *Atp6*,* P* = 0.06), with significant increases in *Cytb* (*P* = 0.04) and *Cox1* (*P* = 0.04). However, we observed no change in the corresponding mitochondrial respiratory chain proteins (i.e., complex I, II, III, IV, and V) in response to maternal exercise (*P* > 0.05, Fig. 4B–G).

### Mitochondrial enzymatic activity in fetal myocardium

**Figure 5:**
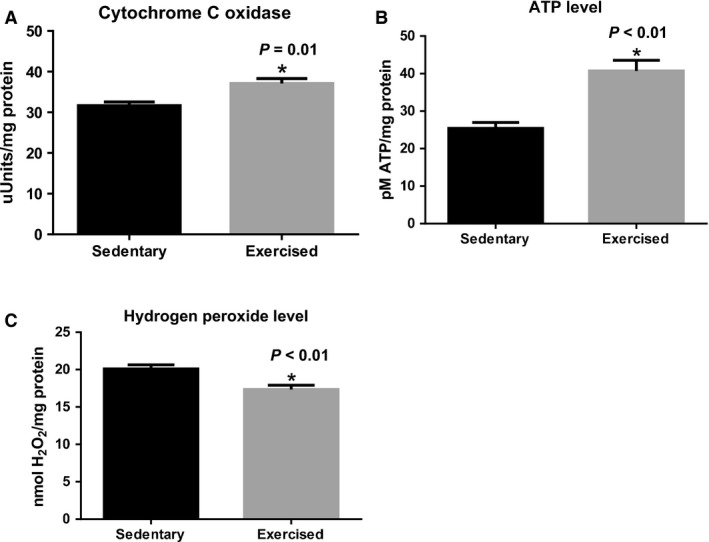
Mitochondrial enzyme activity, ATP, and H_2_O_2_ production. (A) Cytochrome c oxidase activity was significantly increased in the fetal hearts from maternal exercise. (B) ATP production was significantly increased in the fetal hearts from maternal exercise group. (C) Maternal exercise significantly decreased H_2_O_2_ production in the fetal hearts. *n* = 8 per group. Values are expressed as mean ± SEM expressed as fold change relative to the sedentary group. * *P* < 0.05, significantly different from the sedentary group.

We measured cytochrome c oxidase activity, the rate‐limiting enzyme of electron transport chain, and found that maternal exercise significantly increased cytochrome c oxidase activity in the fetal heart (*P* = 0.01, Fig. 5A). ATP is produced largely through oxidative phosphorylation in the mitochondrial electron transport chain. ATP production in fetal myocardium also significantly increased in response to maternal exercise (*P* < 0.01, Fig. 5B). Next, we measured H_2_O_2_ production in the fetal hearts to determine whether maternal exercise induces H_2_O_2_ production. Our result showed that H_2_O_2_ levels were significantly lower in the fetal hearts from exercised dams compared to sedentary dams (*P* < 0.01, Fig. 5C).

## Discussion

To the best of our knowledge, this is the first study to determine the effects of maternal exercise during pregnancy on the expression of mitochondrial gene transcription and translation in the fetal hearts. We demonstrated that increased physical activity through the exposure of a cage‐wheel from day one of pregnancy, in those who were previously sedentary (Fig. 1A and B), did not affect maternal body weight over the course of pregnancy, nor did maternal exercise affect the fetal outcomes, such as number of fetuses per litters, sex distribution, and fetal body weights (Table 2). Aerobic exercise capacity, which typically reflects an individuals' maximal oxygen consumption (*V*O_2max_), is negatively correlated with cardiovascular disease (Blair et al. 1996). A large cohort study showed that aerobic exercise adaptability is largely predetermined by several molecular classifiers including oxidative gene activation (Timmons et al. 2010), which is controlled by mitochondria. Since mitochondrial adaptation commonly occurs in response to exercise training (Judge et al. 2005), we hypothesized that mitochondrial gene expression in fetal myocardium would be upregulated by maternal exercise. In agreement with our hypothesis, several genes associated with mitochondrial biogenesis (i.e., *Nrf1* and *Nrf2* in Fig. 2) and dynamics (*Mfn2* in Fig. 3) were significantly upregulated in fetal myocardium by maternal exercise. Furthermore, mitochondrial enzymatic activity and ATP production were significantly increased, while H_2_O_2_ levels were significantly decreased in the fetal hearts from maternal exercised group compared to those from the sedentary group (Fig. 5).

Mitochondria are dynamic organelles that undergo functional and structural changes in response to various stimuli (El‐Hattab and Scaglia 2016; Verma et al. 2016). Mitochondrial quality control is regulated by mitochondrial biogenesis, mitochondrial dynamics, and mitophagy (Vásquez‐Trincado et al. 2016). Mitochondrial biogenesis is controlled by PGC1*α*, TFAM, NRF‐1 and NRF2 (Dorn et al. 2015). The TFAM and cytochrome c oxidase contain binding sites of NRF1 and NRF2 (Mao and Medeiros 2001). Surprisingly, we found that maternal exercise did not change mRNA levels of *Ppargc1a* and *Tfam*, while it did upregulate *Nrf1* and *Nrf2* (Fig. 2A). Our results confirm previous studies demonstrating temporal regulation of *Nrf1, Nrf2*, and *Tfam*, which increases in *Nrf1* and *Nrf2* precedes upregulation of *Tfam* (Xia et al. 1997; Dorn et al. 2015). Thus, we speculate that upregulation of *Tfam* and *Ppargc1a* may occur in postnatal hearts from exercised dams compared to sedentary dams.

Mitochondrial dynamics has been shown to play an important role in the formation and maintenance of cardiomyocytes (Ishihara et al. 2015). Mitochondrial fusion is regulated by MFN1, MFN2, and OPA1, and mitochondrial fission is regulated by DRP1and FIS1. The balance between fusion and fission is important in mitochondrial DNA homeostasis (Chen et al. 2010). For example, a decreased ratio of MFN2 and DRP1 was found during heart failure (Givvimani et al. 2014), and MFN2 level decreases in pathological cardiac hypertrophy and in failing hearts (Vásquez‐Trincado et al. 2016). Pharmacological inhibition of mitochondrial fission protects the heart from ischemia/reperfusion injury and heart failure (Ong et al. 2010). In addition, CypD has been shown to regulate the mitochondrial permeability transition pore by interacting with mitochondrial transcription factors B1 and B2 (Radhakrishnan et al. 2015) and play an important role in regulating cardiac function (Elrod and Molkentin 2013; Menazza et al. 2013). We did not see large alterations of mitochondrial dynamic genes. Unlike the pathological adaptation, which shows significant downregulation of *Mfn2*(Vásquez‐Trincado et al. 2016), we found *Mfn2* was significantly upregulated in the fetal hearts in response to maternal exercise.

Furthermore, we found most regulation of mitochondrial biogenesis and dynamics occurred at transcriptional levels (i.e., increased mRNA levels) rather than at translational levels (no increase in protein levels) at 17 days of gestation. Our results corroborate previous human and rodent studies showing changes in mitochondrial proliferation and differentiation proceed mainly from the transcriptional level through the fetal developmental stage and translational regulation occurs largely after birth (Cuezva et al. 1997; Alcolea et al. 2006; Minai et al. 2008; Pejznochova et al. 2010). Interestingly, we found cytochrome c oxidase activity, the rate‐limiting enzyme of the electron transport chain, and ATP production were significantly increased in the fetal hearts from exercised dams. The concentration of H_2_O_2_ affects mitochondrial biogenesis and enzyme activity. Modest induction of H_2_O_2_ has been shown to promote mitochondrial biogenesis (Kamble et al. 2013; Park et al. 2013), while a large concentration of H_2_O_2_ induces apoptosis, decreases mitochondrial enzyme activity, and leads to cardiac dysfunction (León et al. 2007; Raisanen et al. 1999). These results suggest that maternal exercise may lead to increased efficiency of mitochondria in the fetal hearts of their offspring.

### Limitations

We could not fully investigate the scope of mitochondrial function since the size of a fetal heart is very small and it is hard to extract adequate RNA and protein, which could be the major limitations of this study. For example, relative amounts of mtDNA to nuclear DNA, typically used to determine mtDNA content (Alcolea et al. 2006; Pejznochova et al. 2010; Park et al. 2013) was not measured. Mitochondrial functional parameters including mitochondrial respiration and membrane potentials, which are typically measured by Clark electrodes, Seahorse analyzer, or Oroboros instrument (Mulligan et al. 2012; Le et al. 2014) were not measured due to technical difficulties of isolating mitochondria or cardiomyocytes from fetal hearts. Mitochondrial structure was not evaluated by electron microscopy analysis. Upcoming studies will investigate aforementioned parameters to better understand the role of maternal exercise on fetal mitochondrial function. Nonetheless, our results demonstrate that maternal exercise could transfer the positive mitochondrial phenotype to fetal hearts by increases in enzyme activity (Maj et al. 2011), ATP production, and several genes regulating mitochondrial biogenesis. Future studies are needed to confirm whether upregulation of mitochondrial gene expression induces increased protein levels after birth and enhances mitochondrial function. Previous studies demonstrate that maternal exercise during pregnancy has positive effects on glucose metabolism in adult offspring even though offspring were raised identically after weaning (Carter et al. 2013; Stanford et al. 2015). These previous studies imply that fetal adaptation due to maternal exercise may persist in later life when metabolic disturbances often occur with the aging process. It would be of great interest to investigate whether the offspring from exercised mothers are less susceptible to stress in adulthood compared to the offspring from sedentary mothers. Further, it will be necessary to investigate whether fetal conditioning by maternal exercise can induce the potential long‐term cardiovascular benefits for the offspring and attenuate cardiac dysfunction when a cardiac insult, such as ischemia/reperfusion injury, is given to the heart. Although more work should be done in this area, we believe our work has significant potential to add to the current research base on the impact of maternal exercise on fetal programming.

In summary, we have demonstrated that maternal exercise initiated at day one of gestation is not detrimental to the fetus (i.e., the number of fetuses per litters, sex distribution, and fetal body weight were all similar between groups), but rather beneficial by activating several mitochondrial genes and enzyme activity in the fetal hearts. Mitochondrial dysfunction is highly linked to cardiomyopathy in children (Holmgren et al. 2003; Scaglia et al. 2004; El‐Hattab and Scaglia 2016), so upregulating mitochondrial biogenesis and dynamics at the developmental stage could be considered the earliest intervention for preventing cardiovascular diseases in later life.

## Conflict of Interest

No conflicts of interest, financial or otherwise, are declared by the authors.
